# *In Vivo* Gene Essentiality and Metabolism in Bordetella pertussis

**DOI:** 10.1128/mSphere.00694-18

**Published:** 2019-05-22

**Authors:** Laura A. Gonyar, Patrick E. Gelbach, Dennis G. McDuffie, Alexander F. Koeppel, Qing Chen, Gloria Lee, Louise M. Temple, Scott Stibitz, Erik L. Hewlett, Jason A. Papin, F. Heath Damron, Joshua C. Eby

**Affiliations:** a Department of Pediatrics, University of Virginia, Charlottesville, Virginia, USA; b Department of Medicine, Division of Infectious Diseases and International Health, University of Virginia, Charlottesville, Virginia, USA; c Department of Medicine, University of Virginia, Charlottesville, Virginia, USA; d Department of Biomedical Engineering, University of Virginia, Charlottesville, Virginia, USA; e Center for Biologics Evaluation and Research, Food and Drug Administration, Silver Spring, Maryland, USA; f Department of Integrated Science and Technology, James Madison University, Harrisonburg, Virginia, USA; g Department of Biochemistry and Molecular Genetics, University of Virginia, Charlottesville, Virginia, USA; h Department of Microbiology, Immunology and Cell Biology, West Virginia University, Morgantown, West Virginia, USA; i Vaccine Development Center, West Virginia University Health Sciences Center, Morgantown, West Virginia, USA; University of Kentucky

**Keywords:** *Bordetella*, *Bordetella pertussis*, Tn-seq, gene essentiality, *in vivo*, metabolism

## Abstract

Bordetella pertussis is the causative agent of whooping cough, a serious respiratory illness affecting children and adults, associated with prolonged cough and potential mortality. Whooping cough has reemerged in recent years, emphasizing a need for increased knowledge of basic mechanisms of B. pertussis growth and pathogenicity. While previous studies have provided insight into *in vitro* gene essentiality of this organism, very little is known about *in vivo* gene essentiality, a critical gap in knowledge, since B. pertussis has no previously identified environmental reservoir and is isolated from human respiratory tract samples. We hypothesize that the metabolic capabilities of B. pertussis are especially tailored to the respiratory tract and that many of the genes involved in B. pertussis metabolism would be required to establish infection *in vivo*. In this study, we generated a diverse library of transposon mutants and then used it to probe gene essentiality *in vivo* in a murine model of infection. Using the CON-ARTIST pipeline, 117 genes were identified as conditionally essential at 1 day postinfection, and 169 genes were identified as conditionally essential at 3 days postinfection. Most of the identified genes were associated with metabolism, and we utilized two existing genome-scale metabolic network reconstructions to probe the effects of individual essential genes on biomass synthesis. This analysis suggested a critical role for glucose metabolism and lipooligosaccharide biosynthesis *in vivo*. This is the first genome-wide evaluation of *in vivo* gene essentiality in B. pertussis and provides tools for future exploration.

**IMPORTANCE** Our study describes the first *in vivo* transposon sequencing (Tn-seq) analysis of B. pertussis and identifies genes predicted to be essential for *in vivo* growth in a murine model of intranasal infection, generating key resources for future investigations into B. pertussis pathogenesis and vaccine design.

## Abstract (precis)

Our study describes the first *in vivo* transposon sequencing (Tn-seq) analysis of B. pertussis and identifies genes predicted to be essential for *in vivo* growth in a murine model of intranasal infection, generating key resources for future investigations into B. pertussis pathogenesis and vaccine design.

## INTRODUCTION

Whooping cough, caused by the Gram-negative bacterium Bordetella pertussis, is a serious respiratory illness in children and adults that can be fatal (1). Despite widespread vaccination, whooping cough has reemerged in recent years, reinvigorating research into B. pertussis pathogenesis and vaccine design (2). Humans are the only natural host for B. pertussis, but animal models, including baboons and mice (3–6), have been developed to facilitate study. Compared with other *Bordetellae*, the B. pertussis genome is smaller with more pseudogenes (9.4% of the genome) and substantial rearrangements mediated by insertion elements (7). This restricted genetic content is thought to be a result of adaptation to the human host (7); however, the genetic elements and changes that facilitate host specificity have not been identified.

Some insight into B. pertussis-host interaction has been gained by single gene mutations and transposon mutagenesis *in vitro* (8–10). Select mutants with altered virulence phenotypes *in vitro* have subsequently been evaluated for the effect of mutation on B. pertussis survival *in vivo*, identifying virulence genes important for infection (11, 12). More recently, high-throughput transposon sequencing (Tn-seq) has been used to identify genes essential for *in vitro* growth of B. pertussis (13). Unlike traditional transposon mutagenesis, which is based on the isolation of individual mutant clones in a transposon library, Tn-seq is based on sequencing of a pool of clones as a community. Because all members of the population are competing for the same limited nutrients, metabolism-associated genes are most often identified by Tn-seq, and Fyson et al. utilized Tn-seq to validate a computational model of B. pertussis metabolism *in vitro* (13).

Because metabolism-associated genes are central to host-pathogen specificity, we used Tn-seq to probe *in vivo* gene essentiality in B. pertussis. We generated a transposon library with high insertion coverage and measured growth *in vitro* and *in vivo* using an intranasal model of murine infection. These studies reveal novel observations about B. pertussis metabolism, gene regulation, and metabolism-associated virulence factors *in vitro* and within the host environment.

## RESULTS

### Construction and validation of Tn library.

**FIG 1:**
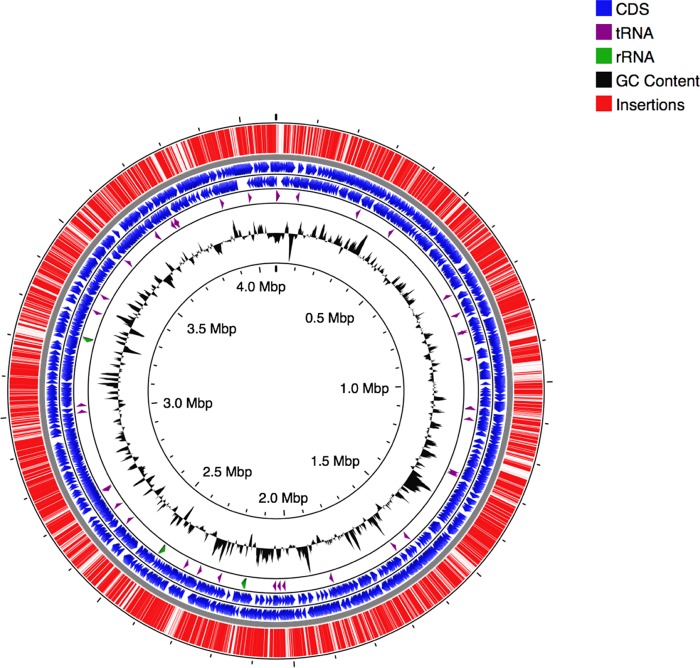
B. pertussis transposon library. A map of insertions (red) and their distribution across the genome was generated by CGView (16). Coding regions of genes (coding sequences [CDS]) are shown in blue and separated by strand. Locations of tRNA and rRNA are shown in purple and green, respectively. GC content is represented in black.

The B. pertussis library was constructed through introduction of the pSAM-Km transposon (14) into B. pertussis strain UT25-*lux* (as described in Text S1 in the supplemental material) by conjugation with an Escherichia coli diaminopimelic acid (DAP) auxotrophic donor strain (15). The library contains approximately 3.0 × 10^6^ clones, determined by enumeration of CFU of the library before harvest from BG plates. High-throughput sequencing on the Illumina Hi-Seq platform revealed that 73.6% of the possible insertion sites (based on the TA insertion site sequence for mariner transposons) and 88.5% of genes were occupied by insertions, which is representative of a highly complex library. Figure 1 shows the distribution of insertions throughout the genome (16). The high insertion coverage occurred despite high GC content of the genome (7, 17).

10.1128/mSphere.00694-18.3 TEXT S1 Supplemental methods. Download Text S1, DOCX file, 0.04 MB. Copyright © 2019 Gonyar et al. 2019 Gonyar et al. This content is distributed under the terms of the Creative Commons Attribution 4.0 International license.

**FIG 2:**
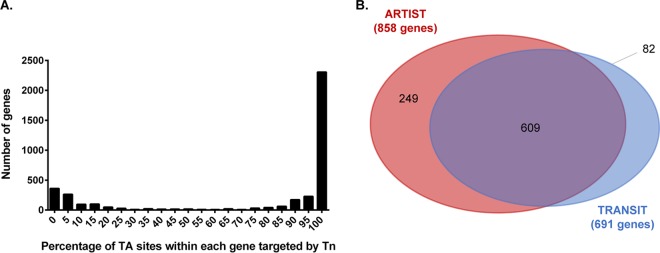
Analysis of *in vitro* gene essentiality using TRANSIT and ARTIST. (A) Histogram showing the TA site insertion saturation per gene. (B) A Venn diagram of genes identified as essential by ARTIST (red) and TRANSIT (blue). A total of 609 genes were identified by both analysis pipelines (purple).

High- and low-density libraries can be distinguished by the separation between peaks on a histogram plotting the percentage of potential insertion sites occupied per gene (Fig. 2A); a more-dense library is characterized by a distinct and greater magnitude difference between peaks (18). The greatest number of genes with a low percentage of TA site occupation is considered the essential gene peak, and the genes with the highest percentage of TA site occupation form the second, nonessential peak. As demonstrated in Fig. 2A, there is a clear separation between the two peaks, which is consistent with a well-saturated library.

Tn-seq data (from samples grown *in vitro* on BG agar) were analyzed using two analysis programs, TRANSIT (19) and ARTIST (20), that both utilize a hidden Markov model (HMM). In Tn-seq analysis, HMMs identify the most probable essentiality state of a region by assessing a sequence of TA sites and incorporating information from neighboring TA sites in order to improve the accuracy of the essentiality determination (21). These programs classify genes as essential based on the essentiality classification of regions within a gene. Classification of a gene as essential presumes that gene loss is associated with an impairment of growth that can be inferred by the number of transposon insertions present in that gene. Using these two HMM-based methods, we generated a list of 609 genes (Fig. 2B; see also Table S2 in the supplemental material) that were classified as essential by both programs and are required for efficient *in vitro* growth (Text S1).

10.1128/mSphere.00694-18.4 TABLE S1 Genes identified as essential by conservative analysis. In this conservative method, only genes with no TA site hits in the first 95% of gene were considered essential. Based on five input samples grown on BG media, the analysis identified 400 essential genes (including tRNAs and rRNAs). Download Table S1, XLSX file, 0.01 MB. Copyright © 2019 Gonyar et al. 2019 Gonyar et al. This content is distributed under the terms of the Creative Commons Attribution 4.0 International license.

10.1128/mSphere.00694-18.5 TABLE S2 Full list of genes identified by ARTIST and TRANSIT. This list includes lists of genes identified individually by ARTIST and TRANSIT as well as the list of 609 genes identified by both programs. It also includes domain essential genes identified by ARTIST. Download Table S2, XLSX file, 0.05 MB. Copyright © 2019 Gonyar et al. 2019 Gonyar et al. This content is distributed under the terms of the Creative Commons Attribution 4.0 International license.

We further analyzed the set of genes classified as essential by both TRANSIT and ARTIST, presuming that genes within this combined set were more likely than either set individually to represent genes that are truly essential *in vitro*. Identification of multiple essential genes within the same metabolic pathway would suggest the importance of that pathway for B. pertussis metabolism and viability. To organize the data, we manually added KEGG pathway classifications to 499 essential genes, which for many genes resulted in more than one annotation per gene (Table S3). The remaining 110 essential genes lacked an identified pathway classification. Mapping to KEGG pathways revealed information about essential metabolic pathways under our growth conditions (Table S3 and Fig. S1). The most represented categories were biosynthesis of secondary metabolites, biosynthesis of antibiotics, ribosome, microbial metabolism in diverse environments, biosynthesis of amino acids, and metabolism of cofactors and vitamins.

10.1128/mSphere.00694-18.1 FIG S1 Representation of *in vitro* (thick black lines) and *in vivo* (red lines) essential genes within the network of bacterial metabolic processes. Colored boxes indicate the approximate locations of selected metabolic reactions associated with a related end product or substrate, including lipooligosaccharide synthesis (green), pentose phosphate (violet), glycolysis and gluconeogenesis (blue), TCA cycle (orange), amino acid synthesis (dark gray), vitamin and cofactor synthesis (yellow), and lipid metabolism (light gray). The figure was created using the KEGG Mapper function on the KEGG database. Download FIG S1, TIF file, 2.5 MB. Copyright © 2019 Gonyar et al. 2019 Gonyar et al. This content is distributed under the terms of the Creative Commons Attribution 4.0 International license.

10.1128/mSphere.00694-18.6 TABLE S3 KEGG pathway assignments for *in vitro* essential genes. Download Table S3, XLSX file, 0.03 MB. Copyright © 2019 Gonyar et al. 2019 Gonyar et al. This content is distributed under the terms of the Creative Commons Attribution 4.0 International license.

**FIG 3:**
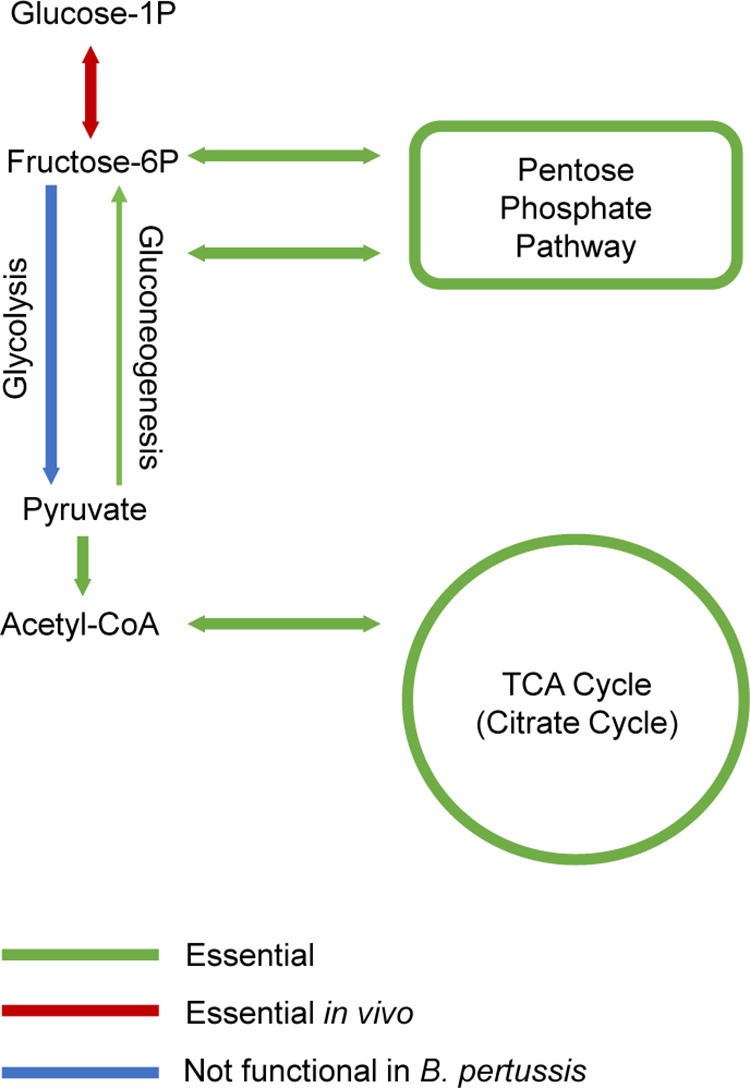
Carbon metabolism in B. pertussis. Pathways with all or many genes identified as essential for *in vitro* growth are shown in green. Genes involved in the interconversion of glucose-1P and fructose-6P were classified as conditionally essential *in vivo*, and this reaction is shown in red.

Multiple genes within the gluconeogenesis and pentose phosphate pathways were classified as essential (Fig. 3), supporting prior findings that these pathways are present and functional in B. pertussis (7). Originally, the tricarboxylic acid (TCA) cycle in B. pertussis was considered nonfunctional despite the observation that the genome contains all required genes (7, 22). Recently, the enzymatic activities thought to be absent (citrate synthase, aconitase, and isocitrate dehydrogenase) in B. pertussis were shown to be present (23). In support of these findings, our data indicated that genes encoding citrate synthase and isocitrate dehydrogenase are essential *in vitro* in our screen, as well as the majority (all but two) of the remaining genes assigned to reactions within the tricarboxylic acid cycle (Fig. 3). The two nonessential genes (*BP2014* and *BP2021*) are annotated as aconitate hydratases (responsible for interconversion of citrate and isocitrate), and these genes may be functionally redundant. Together, these data suggest not only a functional TCA cycle but that the TCA cycle is critical for growth under our *in vitro* growth conditions on BG agar.

**FIG 4:**
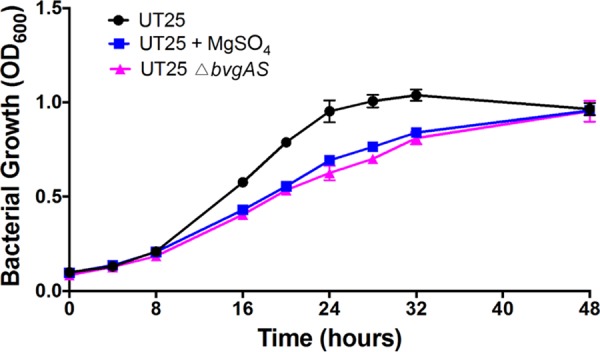
Growth curve of BvgAS-active and BvgAS-inactive B. pertussis UT25. Wild-type (black) and △*bvgAS* B. pertussis UT25 (pink) were grown in SSM as described in Materials and Methods. To modulate wild-type B. pertussis UT25 to a BvgAS-inactive state, 40 mM MgSO_4_ was added to all passages in SSM (blue). Bacterial growth was determined by the optical density at 600 nm (OD_600_).

Unexpectedly, the Bvg two-component system (*bvgAS*) was computationally classified as essential for *in vitro* growth by our analysis by TRANSIT and ARTIST. However, *bvgAS* did tolerate a small number of insertions (three insertions in *bvgS* and one insertion in *bvgA*, all at distinct TA sites) and is not identified as essential by stringent analysis (24). BvgAS has been characterized in many strains of *Bordetellae*, including strains of B. pertussis, and these strains are able to tolerate mutations within the *bvgAS* genes, suggesting that it is not truly essential for *in vitro* growth. To confirm that *bvgAS* could be deleted in the strain used for this study, we generated B. pertussis UT25 Δ*bvgAS*. This strain grows under the conditions that the library was generated (static growth on BG agar at 37°C), suggesting that *bvgAS* are not true essential genes. One explanation is that lacking BvgAS was disadvantageous for growth under our conditions. We tested the hypothesis by comparing the growth of B. pertussis UT25 Δ*bvgAS* to the growth of wild-type B. pertussis UT25 under both modulating (active BvgAS) and nonmodulating (inactive BvgAS) conditions. B. pertussis UT25 exhibited a growth defect when BvgAS was inactive, either genetically or through chemical modulation with MgSO_4_ (Fig. 4), supporting the hypothesis that lacking *bvgAS* was disadvantageous for growth. It is important to note that these growth studies were performed in Stainer-Scholte medium, a standard, defined liquid growth medium for B. pertussis, rather than on BG agar, which was used to generate the library. Demonstration of a BvgAS-dependent growth defect provides a potential explanation for identification of *bvgAS* as essential in our screen, but other explanations for this observation exist, highlighting the limitations of Tn-seq analysis for determining gene essentiality.

### Conditional essentiality *in vivo*.

**FIG 5:**
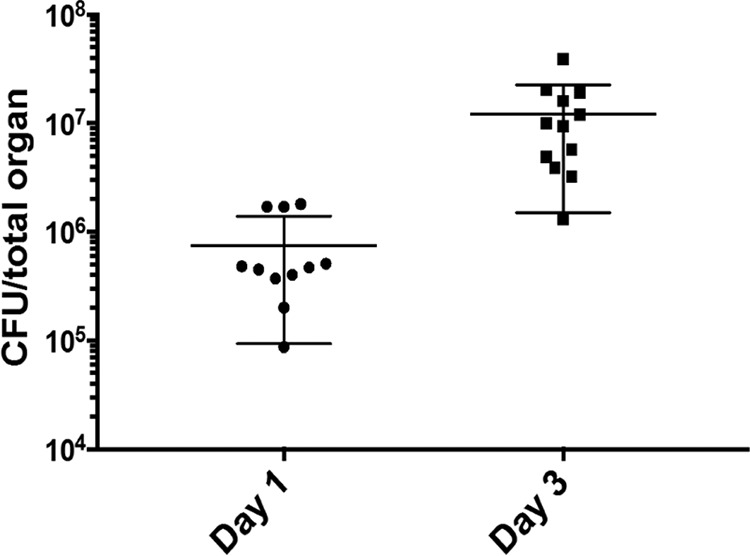
Bacterial burden in organs of B. pertussis*-*infected mice. The lungs and trachea of infected mice were removed and homogenized at the indicated time point, and the homogenates were diluted and plated, and the bacteria were enumerated to determine CFU/organ. Each point represents the total bacteria enumerated from the combined lung and trachea from one animal (*n* = 11 at day 1 and *n* = 12 at day 3). The means ± standard deviations (error bars) are shown.

To determine which genes contribute to infection, CD1 mice were intranasally infected with approximately 5 × 10^6^ CFU of the library cultivated on BG agar. The mice were euthanized at either day 1 or day 3 postinfection, and B. pertussis conditional gene essentiality was determined at each of these time points. The average bacterial burden in combined lung and trachea samples at day 1 was 7.4 × 10^5^ CFU/organ, and at day 3, it was 1.2 × 10^7^ CFU/organ (Fig. 5), consistent with published work using this strain of mouse and a similar inoculum (25). The entire organ homogenate was plated on BG agar, and then bacterial colonies were harvested after 3 days of growth, genomic DNA was isolated, and samples were prepared for high-throughput sequencing by Illumina HiSeq platform.

We used ARTIST (20) for *in vitro* to *in vivo* comparisons, which utilizes simulation-based normalization followed by initial analysis by Mann-Whitney U tests and further refinement using a hidden Markov model. Genes that are classified as essential for *in vitro* growth are excluded from *in vivo* analysis, as clones with insertions in these genes are absent from our library. The ARTIST pipeline includes analysis to determine whether a bottleneck is present under the experimental conditions that could influence essentiality classifications despite normalization. When the bottleneck simulation was run on our *in vitro* data using parameters from our *in vivo* experimental conditions, the false-positive rates calculated for each of the three input samples were 2.9, 5.3, and 4.0% with standard deviations of 0.08, 0.12, and 0.08%, respectively. This result indicates an estimated false-positive rate of about 4% for each individual *in vivo* sample. In order to reduce the chance that we would falsely designate a gene as essential, we utilized the following consensus method. After ARTIST computationally classified each individual *in vivo* sample by comparison to the respective *in vitro* sample, we examined the ARTIST-derived gene essentiality for each of the 11 or 12 replicates of each gene at each time point (Table S4). If a replicate was classified as essential, the gene associated with that replicate would earn a point. The sum of the replicate points was the consensus score. We designated a gene as essential based on the consensus score. As anticipated, increasing the stringency, by requiring a greater consensus score in order to designate a gene as essential, resulted in designation of a smaller number of genes as conditionally essential (Fig. S2). We designated a gene as conditionally essential if >50% of the replicates (a score of 6) for that gene were computationally classified as essential as previously described (20). Thus, genes designated as essential by this consensus method reduce false-positive essentiality designations attributable to the effect of the bottleneck on computational gene classification (20).

10.1128/mSphere.00694-18.2 FIG S2 Number of genes classified as essential decreases as stringency in the consensus score increases. Download FIG S2, TIF file, 0.4 MB. Copyright © 2019 Gonyar et al. 2019 Gonyar et al. This content is distributed under the terms of the Creative Commons Attribution 4.0 International license.

10.1128/mSphere.00694-18.7 TABLE S4 Table used for consensus determination for *in vivo* essential genes. For each sequencing sample (derived from one infected animal), ARTIST classified each gene or intergenic region as essential (designated as 1) or nonessential (designated as 0). The consensus score was calculated by taking the sum of this essentiality classifications; for example, a gene that was identified as essential in seven animals would have a consensus score of 7. Download Table S4, XLSX file, 0.6 MB. Copyright © 2019 Gonyar et al. 2019 Gonyar et al. This content is distributed under the terms of the Creative Commons Attribution 4.0 International license.

**FIG 6:**
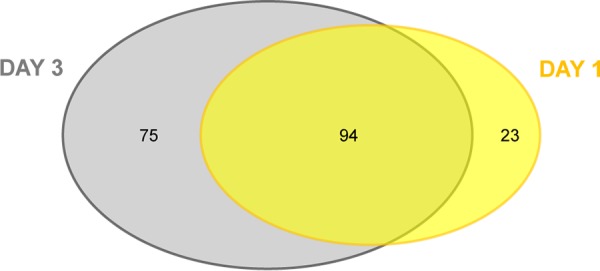
B. pertussis genes that are conditionally essential *in vivo*. CON-ARTIST was used for this analysis; 117 essential genes identified at 1 day postinfection are shown in yellow, and 169 genes identified as essential at 3 days postinfection are shown in gray.

We chose to perform these analyses at both day 1 and day 3 postinfection to determine whether gene essentiality differed as infection progressed. Three days postinfection is at or near the peak of bacterial burden (25). We hypothesized that the number of essential genes would increase at day 3 compared to day 1 as the bacterial population continued to be exposed to selection pressure. We identified 117 genes as conditionally essential at day 1 postinfection and 169 genes as conditionally essential at day 3 postinfection, with 94 genes shared between the two time points (Fig. 6 and Table S5). When all the genes identified at either day 1 or day 3 postinfection are included, 192 genes are identified as conditionally essential (Table S5).

10.1128/mSphere.00694-18.8 TABLE S5 Genes identified by ARTIST as essential on day 1 and day 3 postinfection. Genes had a consensus score greater than or equal to 6 for day 1 or 7 for day 3, which corresponds to being classified as essential in greater than 50% of the animals. Annotations for genes identified as essential on day 1 and day 3 postinfection are also included. Annotations were based on KEGG pathway assignments. Download Table S5, XLSX file, 0.03 MB. Copyright © 2019 Gonyar et al. 2019 Gonyar et al. This content is distributed under the terms of the Creative Commons Attribution 4.0 International license.

### B. pertussis *in vivo* metabolism.

Very few prototypical virulence factors were identified, and this was anticipated based on the methodology of Tn-seq. Clones in the library with mutations of genes encoding secreted factors (for example, *cyaA* encoding adenylate cyclase toxin and *ptxA* encoding pertussis toxin) are complemented by other library clones within the population and are not identified in this screen. B. pertussis carries genes encoding an arsenal of adhesins, including Fim2, Fim3, and filamentous hemagglutinin that are included in the acellular vaccine, and not identifying these factors is expected, as their functions may be redundant in our model of infection. A recent study utilized RNA-seq to characterize the BvgAS regulon, including describing metabolic genes under BvgAS control (26). BvgAS is well characterized for its role in regulation of B. pertussis virulence, and we hypothesized that some metabolic genes identified as essential in our *in vivo* model would be under BvgAS control because of their importance in adaptation to the host nutritional environment. Because BvgAS-activated genes and Bvg-repressed genes have been shown to be expressed during B. pertussis infection in murine models (27), we compared our *in vivo* essential list with known BvgAS-activated and BvgAS-repressed genes (26). Out of the 192 genes identified at day 1 or day 3 postinfection, 7 were positively regulated by BvgAS, and 22 were negatively regulated by BvgAS (Table 1). Most of these 29 genes are associated with metabolism, suggesting a role for BvgAS in coordination of B. pertussis metabolism *in vivo*.

**TABLE 1:** BvgAS regulation of conditionally essential genes *in vivo*

Gene category and locus tag/gene name	Annotation
BvgAS-activated genes	
*BP0790*/*ilvH*	Acetolactate synthase 3 regulatory subunit
*BP1127*	
*BP2930*/*cyoD*	Cytochrome 0 ubiquinol oxidase
*BP2932*/*cyoB*	Ubiquinol oxidase subunit I
*BP2934*	Two-component system response regulator
*BP2935*	Two-component system, histidine kinase
*BP3494*/*brkA*	Serum resistance protein

BvgAS-repressed genes	
*BP0250*	Hypothetical protein
*BP0618*/*lpdA_1*	Dihydrolipoamide dehydrogenase
*BP0619*	Branched-chain amino acid ABC transporter ATP-binding protein
*BP0670*	Acyl-CoA dehydrogenase
*BP0671*	Hypothetical protein
*BP0682*	Putative exported protein
*BP0683*	4,5-Dihydroxyphthalate decarboxylase
*BP0684A*	2Fe-2S ferredoxin
*BP0808*	Hypothetical protein
*BP1631*/*wcbA*	Capsular polysaccharide export protein
*BP1818*/*dadA_1*	d-Amino acid dehydrogenase small subunit
*BP2368*/*prpC*	2-Methylcitrate synthase
*BP2644*	Glycerol-3-phosphate dehydrogenase
*BP3228*/*minD*	Septum site-determining protein
*BP3575*	Hypothetical protein
*BP3671*	Glycosyltransferase family protein
*BP3672*	Hypothetical protein
*BP3679*	TetR family transcriptional regulator
*BP3740*/*colII*	Cytochrome *c* oxidase subunit II
*BP3743*/*ctaD*	Cytochrome *c* oxidase polypeptide I
*BP3744*	Cytochrome *c* oxidase subunit II
*BP3831*	Amino acid ABC transporter substrate-binding protein

The majority of genes required for infection had metabolic functions. Out of the total 192 genes designated as conditionally essential *in vivo*, 117 had one or more KEGG pathway classifications (Table S5 and Fig. S1). Genes associated with transport, biosynthesis of secondary metabolites, and biosynthesis of antibiotics were enriched in our analysis (Table S5 and Fig. S1). Twenty-eight genes annotated as transporters were conditionally essential *in vivo* (Table S5). The specificity of the majority of these transporters is not yet known. We identified *BP3494*, encoding the BrkA autotransporter, as conditionally essential *in vivo*. B. pertussis strains lacking *brkA* are more sensitive to serum *in vitro* and are less virulent in mice (28–30), and our results are consistent with these previous observations. Another autotransporter, the vaccine component pertactin, was not identified, possibly due to the redundancy of autotransporter function. Genes within the *bhu* operon (BP0344-BP0346), encoding a heme iron acquisition system (31), were essential at day 3 but not day 1, consistent with previous literature that suggested that heme becomes more available to B. pertussis later in infection perhaps through B. pertussis-mediated host damage (32).

Genes encoding a quinol oxidase (*cyoABCD*) were conditionally essential *in vivo*. Similarly, these genes were identified in an *in vivo* transposon mutagenesis screen in P. aeruginosa (14). Identification of this complex as important in two very different models of infection with two distinct pathogens may highlight a role of the Cyo terminal oxidase in aerobic respiration *in vivo*, potentially in adaptation to lower oxygen concentrations within the host (33).

### *In silico* analysis of Tn-seq data.

Most of the genes identified in our *in vivo* analysis were involved in metabolic functions, based on current annotation and homology, and we used computational tools to obtain a more complete understanding of each gene’s role in B. pertussis growth. Genome-scale metabolic network reconstructions (GENREs) are computational representations of all the information known about the metabolism of an organism, generated from literature and from genomic, proteomic, and transcriptomic data sets. By mathematically representing the known metabolic genes, enzymes, metabolites, and reactions involved in carrying out the chemical processes in a network, it is possible to relate the genotype and phenotype. Through the use of constraint-based analyses (34, 35), computational predictions of the organism’s metabolic capabilities, including the impact of gene knockouts and of varied medium conditions on growth, allow generation of novel hypotheses.

Two genome-scale metabolic network reconstructions have been recently published for B. pertussis (13, 36), and these models were used as the basis for our calculations. For any model, input nutrients must be defined for simulating biomass production, and we chose components and stoichiometry based on existing defined medium formulations. Biomass production was simulated using each model with an array of nutrient inputs, including unrestricted nutrient availability, two different published formulations of SSM (36, 37), and sputum media (SCFM) (38). BG medium is not defined and cannot be used as an input for modeling biomass production. SSM medium formulations are defined, but they were not originally designed with the goal of simulating the *in vivo* environment and likely are not representative of nutrient availability within the host. SCFM was developed as a chemically defined medium to approximate nutrients available in the respiratory tracts of humans with cystic fibrosis (CF). Although the nutrient composition of CF sputum is different than that of patients affected by pertussis, SCFM is a suitable medium for application to the GENRE and may better represent growth conditions for B. pertussis in the respiratory tract.

The Fyson et al. GENRE (13) and the Branco dos Santos et al. GENRE (36) each generated biomass when grown *in silico* on SCFM media and when provided with unrestricted nutrient availability. Under the SCFM condition, both models indicated lower biomass production yields relative to unrestricted nutrient availability for uptake and export, as would be expected when comparing a defined media where some metabolites are limited or not provided. The Branco dos Santos et al. model indicated generation of biomass with both formulations of SSM as the input media, but the Fyson et al. model, which was tested previously with nutrients provided by charcoal agar, suggested that biomass could not be synthesized on SSM. When single gene deletions were simulated under the SCFM conditions, the Fyson et al. model predicted that 332 of the model’s 796 genes are essential for growth and the Branco dos Santos et al. model predicted that 241 of the model’s 770 genes are essential for growth. The model-predicted essential genes lists were each compared to genes found to be experimentally essential *in vivo* in our Tn-seq analyses (Table S6).

10.1128/mSphere.00694-18.9 TABLE S6 Results of *in silico* analysis using two published GENREs. Download Table S6, XLSX file, 0.03 MB. Copyright © 2019 Gonyar et al. 2019 Gonyar et al. This content is distributed under the terms of the Creative Commons Attribution 4.0 International license.

We used the two GENREs to probe the function of Tn-seq-designated *in vivo* essential genes associated with B. pertussis metabolism using SCFM as the input medium. Based on Tn-seq analysis, two genes encoding products involved in glucose metabolism were essential despite the fact that B. pertussis does not utilize glucose for growth. At both 1 day and 3 days postinfection, *BP3141* and *BP3142*, encoding a phosphoglucomutase and a glucose-6-phosphate isomerase, respectively, were conditionally essential. These enzymes facilitate the conversion of glucose to fructose-6-phosphate, which can then enter either the glycolysis pathway or the pentose phosphate pathway (Fig. 3). Since glycolysis is not functional in B. pertussis, we hypothesized that these enzymes were likely shuttling glucose into the pentose phosphate pathway.

When the genes were deleted *in silico*, model-simulated biomass production was inhibited due to the inability to synthesize lipooligosaccharide (LOS) (Table S6). When genes involved in the pentose phosphate pathway were deleted *in silico*, model-simulated biomass production was similarly inhibited, but outputs derived from other metabolic pathways in addition to LOS biosynthesis were affected as well (Table S6). This result suggested that *BP3141* and *BP3142* were likely contributing to LOS biosynthesis independently of the known LOS biosynthesis precursors generated by the pentose phosphate pathway. In agreement with this finding, West et al. reported that a strain lacking the homolog to *BP3141* in the related organism Bordetella bronchiseptica was less able to survive in a murine model of respiratory infection and exhibited an altered lipopolysaccharide (LPS) profile with no O antigen and a truncated core oligosaccharide (39). Glucose is a component of the outer core *Bordetella* LOS, and we next hypothesized that *BP3141* and *BP3142* are required for generation of glucose-1-phosphate (glucose-1P) as a precursor for LOS outer core synthesis. To address this hypothesis, we investigated whether the genes required for the addition of glucose to the outer core were similarly required for *in vivo* fitness. A UDP-glucose:LOS-beta-1,4-glucosyltransferase adds the first glucose residue to the HepI moiety of LOS. The gene encoding this activity (*BP2329*) has been identified within a novel lipopolysaccharide core biosynthesis gene cluster in B. pertussis (40). *BP2329* is classified by our Tn-seq analysis as conditionally essential *in vivo*, as well as the two other genes within this locus that also result in a truncated LOS (*BP2328* and *BP2330*) (40). Mutation of the remaining gene within this operon, *BP2331*, did not result in a truncated LOS molecule (40) and was not identified as conditionally essential *in vivo* in our analyses. Other genes annotated as having a role in LOS biosynthesis that were assigned as conditionally essential *in vivo* by Tn-seq were *BP2325* and *BP0388*. *BP2325* encodes a putative UDP-glucose:heptosyl-alpha-1,3-glucosyltransferase which adds *N*-acetylglucosamine (a derivative of glucose) to the HepII moiety. *BP0388* is one of the few genes in the major LOS pathway that was not essential *in vitro*, and it encodes phosphoheptose isomerase, which incorporates sedoheptulose-7P into the LOS molecule. Cumulatively, these results suggest a vital role of biosynthesis of full-length LOS in our *in vivo* model and demonstrate how the GENREs aid in interpreting Tn-seq data.

### Correlation of gene essentiality and single nucleotide polymorphism frequency in human isolates of B. pertussis.

By definition, mutations or variants in essential genes of a genome are more likely than variants in nonessential genes to be associated with a growth or fitness defect. We hypothesized that this concept would be applicable to B. pertussis within its natural human host, and we tested whether data on variation in individual genes from human isolates correlated with our gene essentiality data determined by Tn-seq. For this comparison, we utilized the densities of single nucleotide variants (SNVs) within genes from B. pertussis isolates as described previously (41). SNV densities were calculated based on a collection of 343 strains isolated between 1920 and 2010 (41). The essential gene list derived from our data contained the 609 genes that were designated essential by both TRANSIT and ARTIST analysis (Table S2), and the nonessential list included the remaining 3015 genes encoded in the Tohama genome. Based on analysis using an unpaired *t* test with Welch’s correction, there were significantly fewer (*P* < 0.0001) SNVs, both silent and nonsilent, in genes that are essential *in vitro* (mean total SNV density of 0.0009588) versus genes that are not essential *in vitro* (mean total SNV density of 0.001391).

Additionally, we hypothesized that genes that are essential *in vivo* would also be associated with fewer SNVs in this data set than genes identified *in vivo* as nonessential. Genes that are essential *in vitro* are unable to be assessed *in vivo*, and therefore, these genes were excluded from this analysis. For *in vivo* essential genes identified by our screen, we used 192 genes classified as conditionally essential at either day 1 or day 3 postinfection (Table S5) and compared SNV density in those genes to SNV density in genes that are not essential *in vivo* (2,823 genes) using an unpaired *t* test with Welch’s correction. There was no significant difference (*P* = 0.0735) in the total SNV density between essential genes (mean total SNV density of 0.001227) and nonessential genes (mean total SNV density of 0.001402). Nonsilent SNVs are more likely to affect function of a gene product and are therefore more similar to transposon insertion. In contrast to the total SNV density comparison, there were fewer nonsilent SNVs in *in vivo* essential genes (mean SNV density of 0.0006634) compared to nonessential genes (mean SNV density of 0.0008771) (*P* = 0.0023). Altogether, these findings suggest that Tn-seq-derived gene essentiality presented in this study may correlate with gene variation identified in clinical isolates, an interesting finding given the differences between the method of Tn-seq and the nature of variation in the natural host.

## DISCUSSION

This study describes the first *in vivo* Tn-seq analysis of B. pertussis, thus identifying genes predicted to be necessary for wild-type levels of both *in vitro* and *in vivo* growth. These data have been incorporated into two published metabolic models. One is a method for exploring meaningful connections between essential genes, and the other is aimed at improving understanding of essential gene function. We also incorporated data from SNVs of clinical isolates, thus integrating our *in vivo* essentiality predictions with data from human B. pertussis infections.

While many previous studies have investigated B. pertussis metabolism in order to optimize growth *in vitro*, the resulting medium formulations likely do not reflect nutrient availability *in vivo*. Because the evolution of B. pertussis has involved both massive gene loss and a narrowing of its host range, we reasoned that its metabolic activities might be especially tailored to the human respiratory tract and that as a result, many of the genes involved might be critical for establishing infection *in vivo*. Additionally, the syntheses of some known virulence factors depend on intermediates generated by central or secondary metabolic pathways, for example, lipopolysaccharides, exopolysaccharides, siderophores, and quorum-sensing molecules (42). In this sense, any gene that contributes to the fitness and survival of the pathogen may be considered a virulence gene (43). Here, we use Tn-seq and metabolic modeling to establish links between a known virulence factor, LOS, and its metabolic building blocks.

B. pertussis does not grow on typical carbohydrates as the sole carbon source due to an incomplete glycolysis pathway; three required genes (glucokinase, phosphofructokinase, and fructose-1,6-bis-phosphotase) are absent from the genome (7). However, the pathway for gluconeogenesis is apparently fully functional. Our data show that this pathway is essential, as transposon insertions in the genes encoding enzymes that convert pyruvate to fructose-6P were underrepresented in *in vitro* growth conditions. Our *in vivo* analysis also revealed that the genes interconverting fructose-6P and glucose-1P, *BP3141* and *BP3142*, were both essential at day 1 and at day 3 postinfection. As glycolysis is not functional, we hypothesize that the selective advantage conferred by this pathway is in the utilization of glucose to produce full-length lipo-oligosaccharide, as glucose-1P is a component of the LOS outer core. Consistent with this finding is the observation that a mutation in the homolog to *BP3141* in B. bronchiseptica resulted in an altered LPS profile, with no O antigen produced, a truncated core oligosaccharide, and decreased survival in a murine model of respiratory infection (39). This further supports the importance of the *BP3142* phosphoglucomutase in *Bordetellae* and suggests that the role of this enzyme in B. pertussis pathogenesis may be in LOS biosynthesis during infection. Interpretation of these data was aided by *in silico* analysis using GENREs and highlights the utility of these programs in interpreting Tn-seq data sets. Our identification in this study of additional genes involved in the production of mature, full-length LOS provides compelling evidence that this pathway is crucial for B. pertussis survival in the murine respiratory tract.

An unusual finding was the identification of the Bvg two-component system as contributing to wild-type levels of *in vitro* growth under our culture conditions. Mutations in *bvgAS* have been constructed in multiple B. pertussis strains, including a B. pertussis UT25 Δ*bvgAS* strain generated in this study. This strain was able to be generated and grown under the conditions used for the screen, which is validation that these genes are not truly “essential.” However, we found that a B. pertussis UT25 Δ*bvgAS* strain exhibited a growth defect in comparison to a wild-type parental strain, and this growth defect is replicated by chemical modulation to a Bvg inactive state with MgSO_4_. We speculate that this growth defect was likely further exacerbated under competitive growth conditions in a pool of clones possessing wild-type *bvgAS* alleles. Other potential explanations for this finding include the potential for functionally or structurally guarded genes that do not permit transposon insertion in Bvg such as extensive binding proteins or a three-dimensional structure of the gene (44). Although the finding with *bvgAS* serves as evidence that some of the genes identified in our screen, more likely through the hidden Markov model analyses than the more stringent analyses, are not true essential genes, these genes contribute to efficient growth under our conditions and therefore are important for exploration.

We asked whether essential genes identified by Tn-seq predict which genes in B. pertussis are subject to variation and tested this question by comparing *in vivo* Tn-seq-based gene essentiality to SNVs identified in 343 strains of B. pertussis collected between 1920 and 2010 (41). We found that *in vitro* essential genes had a lower total and nonsilent SNV density than *in vitro* nonessential genes. Genes that were conditionally essential *in vivo* also had a lower nonsilent SNV density than genes that were not essential *in vivo*. These data suggest that genes identified in the *in vitro* and *in vivo* studies as essential are associated with gene variation identified in clinical isolates from human infection.

There are caveats to this suggestion that the comparison of *in vivo* essential genes to SNV data supports a connection of the Tn-seq essentiality assignments to human infection. The screen was performed in an intranasal murine infection model, which differs from human infection with B. pertussis and does not replicate the clinical features of pertussis. The method of inoculation and burden of infection may result in different selective pressure due to anatomy, nutrient availability, and different inflammatory responses among other aspects. The early time points of the Tn-seq *in vivo* studies likely include different selective pressures than those to which human isolates are subjected in the first several weeks of human infection prior to cultures being obtained. Nonetheless, we expect that some of the selective pressures and growth requirements in humans and mice over these differing times are comparable, particularly in the case of metabolic genes included in the Tn-seq data set. In fact, the abundance of metabolic genes favors the association between Tn-seq and SNV analysis, as virulence factors vary more than nonvirulence factors (45).

In addition to host- and infection-related differences between Tn-seq data and SNV data, there are fundamental methodologic differences between these data. In Tn-seq, a complex library of clones containing unique insertions located across the genome is created simultaneously in a single isolate and then subjected to pressure. The forward introduction of variation is therefore presumed to be equal across the genome except for experimental biases introduced by the transposon’s (e.g., TA site preference) ability to insert into structurally guarded genes (e.g., protein binding regions or H-NS regions [44]) and the *in vitro* conditions under which the library was generated. It is essentially also comprehensive, if saturating Tn mutagenesis is performed. SNVs, on the other hand, accumulate over time, both randomly and in response to host selection, their introduction is far from comprehensive, and their detection is limited by the degree of sampling of the genome, allelic frequency, and number of samples interrogated (46, 47). The effects of SNVs, with the exception of nonsense and frameshift mutations, on gene function will be impossible to predict in the vast majority of cases. Transposon insertion into a gene, on the other hand, will usually disrupt gene function.

Presumably, increased time of the Tn-seq pool within the host will subject it to prolonged and/or additional pressures, which may alter the spectrum of genes identified as essential *in vivo*. Balancing this, prolonged residence in the host may result in fewer bacteria recovered, resulting in loss of diversity in the Tn-seq output pool, due to random loss of subpopulations stochastically. Robust sequencing and analysis require sufficient numbers of bacteria in order to maintain statistical and analytical power. With a smaller number of bacteria recovered, the number of replicates would have to be increased substantially for statistical power. Therefore, the time point of 3 days, which is at or near the peak of bacterial burden (25) was chosen. Bacteria recovered from the host at day 1 postinfection have been subjected to limited host pressure, and output pool analysis at this earlier time point could logically be expected to show fewer essential genes than at day 3 postinfection. Furthermore, analysis of day 1 or day 3 output pools is expected to be more indicative of early growth requirements in the host and/or of selective pressures from innate, rather than adaptive, immune mechanisms. Future studies will be required to assess selective pressures experienced at later time points and may be challenging due to decreased bacterial recovery.

In this study, we generated a diverse library of transposon mutants and used this library to probe gene essentiality *in vivo* in a murine model of intranasal infection. This screen generated a large data set that will be a valuable resource for future investigations into B. pertussis pathogenesis to support the design of improved vaccines. It also enabled novel observations about metabolism and gene regulation *in vitro* and *in vivo*. Integration of these data with other published reports and available metabolic models provided a more comprehensive understanding of metabolism and gene essentiality in this pathogen that has undergone genome condensation and reduction of its host range during its evolution. Understanding B. pertussis metabolism and gene essentiality *in vivo* may also be used to direct the design of growth media that more accurately reflect *in vivo* growth and that may therefore enable identification of new vaccine antigen candidates.

## MATERIALS AND METHODS

Additional methods are included in Text S1 in the supplemental material.

### Ethics statement.

All animal work was approved by the University of Virginia Institutional Animal Care and Use Committee protocol 4004.

### Construction of library.

B. pertussis strain UT25-lux (Text S1) was grown as indicated on BG and then passaged into SSM for growth for 20 to 24 h at 35.5°C shaking. The culture was diluted to an optical density at 600 nm (OD_600_) of 0.08 and grown for 18 to 20 h to an OD_600_ of 0.7 to 0.8. E. coli strain RHO3 (15) was grown shaking at 35.5°C for 16 to 18 h in the presence of DAP. Cultures of B. pertussis and E. coli were washed to remove antibiotics, combined in SSM (50 to 100 μl), and incubated statically on BG+MgSO_4_+DAP for 18 to 24 h at 37°C. The mixed mating was then harvested, washed once, and plated on BG+kanamycin in the absence of DAP. Colonies were visible after 3 days of growth at 37°C. Transposon insertion was verified by positive PCR for the kanamycin cassette and negative PCR for the pSAM backbone, suggesting integration of the transposon into chromosomal DNA and loss of the pSAM-Km plasmid. Southern blot analysis indicated that most clones had a single insertion site.

### *In vitro* and *in vivo* screening of library.

For preparation of bacterial inocula, 1 × 10^8^ CFU of library was plated on four large plates, grown for 3 days on BG+Kan, collected, washed, and replated on fresh BG for 2 days. The colonies were harvested by swabbing, washed, and diluted to obtain the desired dose. Four-week-old CD1 mice (Charles River) were infected intranasally with 20 μl of PBS containing approximately 5 × 10^6^ CFU. Genomic DNA from the remainder of the inoculum was prepared and used as the input sample for comparative analysis and the BG-grown sample for *in vitro* analysis. Mice were euthanized at day 1 (*n* = 11) or day 3 (*n* = 12), and lungs and trachea were extracted, weighed, pooled, and homogenized. Organ homogenates were serially diluted and plated to determine bacterial load; the remainder of the homogenized sample was plated on BG, grown for 2 days, and collected for preparation of genomic DNA. Genomic DNA was prepared following the manufacturer’s instructions (Wizard genomic DNA purification kit; Promega).

### Accession number(s).

The mapped reads are available at the Sequence Read Archive (SRA) under BioProject accession number PRJNA542053 and SRA accession number SRP197242.

10.1128/mSphere.00694-18.10 TABLE S7 Oligonucleotide sequences. Download Table S7, XLSX file, 0.01 MB. Copyright © 2019 Gonyar et al. 2019 Gonyar et al. This content is distributed under the terms of the Creative Commons Attribution 4.0 International license.

## References

[1] Jakinovich A, Sood SK 2014 Pertussis: still a cause of death, seven decades into vaccination. Curr Opin Pediatr 26:597–604. doi:10.1097/MOP.0000000000000139. 25136948

[2] Warfel JM, Edwards KM 2015 Pertussis vaccines and the challenge of inducing durable immunity. Curr Opin Immunol 35:48–54. doi:10.1016/j.coi.2015.05.008. 26091979

[3] Warfel JM, Beren J, Kelly VK, Lee G, Merkel TJ 2012 Nonhuman primate model of pertussis. Infect Immun 80:1530–1536. doi:10.1128/IAI.06310-11. 22252879 PMC3318410

[4] Warfel JM, Beren J, Merkel TJ 2012 Airborne transmission of *Bordetella pertussis*. J Infect Dis 206:902–906. doi:10.1093/infdis/jis443. 22807521 PMC3501154

[5] Elahi S, Holmstrom J, Gerdts V 2007 The benefits of using diverse animal models for studying pertussis. Trends Microbiol 15:462–468. doi:10.1016/j.tim.2007.09.003. 17920273

[6] Mills KHG, Gerdts V 2014 Mouse and pig models for studies of natural and vaccine-induced immunity to *Bordetella pertussis*. J Infect Dis 209 Suppl 1:S16–S19. doi:10.1093/infdis/jit488. 24626866

[7] Parkhill J, Sebaihia M, Preston A, Murphy LD, Thomson N, Harris DE, Holden MTG, Churcher CM, Bentley SD, Mungall KL, Cerdeño-Tárraga AM, Temple L, James K, Harris B, Quail MA, Achtman M, Atkin R, Baker S, Basham D, Bason N, Cherevach I, Chillingworth T, Collins M, Cronin A, Davis P, Doggett J, Feltwell T, Goble A, Hamlin N, Hauser H, Holroyd S, Jagels K, Leather S, Moule S, Norberczak H, O’Neil S, Ormond D, Price C, Rabbinowitsch E, Rutter S, Sanders M, Saunders D, Seeger K, Sharp S, Simmonds M, Skelton J, Squares R, Squares S, Stevens K, Unwin L, Whitehead S, Barrell BG, Maskell DJ 2003 Comparative analysis of the genome sequences of *Bordetella pertussis*, *Bordetella parapertussis* and *Bordetella bronchiseptica*. Nat Genet 35:32–40. doi:10.1038/ng1227. 12910271

[8] Merkel TJ, Stibitz S 1995 Identification of a locus required for the regulation of bvg-repressed genes in *Bordetella pertussis*. J Bacteriol 177:2727–2736. doi:10.1128/jb.177.10.2727-2736.1995. 7751282 PMC176943

[9] Knapp S, Mekalanos JJ 1988 Two trans-acting regulatory genes (*vir* and *mod*) control antigenic modulation in *Bordetella pertussis*. J Bacteriol 170:5059–5066. doi:10.1128/jb.170.11.5059-5066.1988. 2903140 PMC211571

[10] Weiss AA, Melton AR, Walker KE, Andraos-Selim C, Meidl JJ 1989 Use of the promoter fusion transposon Tn*5 lac* to identify mutations in *Bordetella pertussis* vir-regulated genes. Infect Immun 57:2674–2682. 2569447 10.1128/iai.57.9.2674-2682.1989 PMC313511

[11] Goodwin MS, Weiss AA 1990 Adenylate cyclase toxin is critical for colonization and pertussis toxin is critical for lethal infection by *Bordetella pertussis* in infant mice. Infect Immun 58:3445–3447. 2401570 10.1128/iai.58.10.3445-3447.1990 PMC313675

[12] Marr N, Oliver DC, Laurent V, Poolman J, Denoël P, Fernandez RC 2008 Protective activity of the *Bordetella pertussis* BrkA autotransporter in the murine lung colonization model. Vaccine 26:4306–4311. doi:10.1016/j.vaccine.2008.06.017. 18582518

[13] Fyson N, King J, Belcher T, Preston A, Colijn C 2017 A curated genome-scale metabolic model of *Bordetella pertussis* metabolism. PLoS Comput Biol 13:e1005639. doi:10.1371/journal.pcbi.1005639. 28715411 PMC5553986

[14] Skurnik D, Roux D, Aschard H, Cattoir V, Yoder-Himes D, Lory S, Pier GB 2013 A comprehensive analysis of *in vitro* and *in vivo* genetic fitness of *Pseudomonas aeruginosa* using high-throughput sequencing of transposon libraries. PLoS Pathog 9:e1003582. doi:10.1371/journal.ppat.1003582. 24039572 PMC3764216

[15] López CM, Rholl DA, Trunck LA, Schweizer HP 2009 Versatile dual-technology system for markerless allele replacement in *Burkholderia pseudomallei*. Appl Environ Microbiol 75:6496–6503. doi:10.1128/AEM.01669-09. 19700544 PMC2765137

[16] Stothard P, Wishart DS 2005 Circular genome visualization and exploration using CGView. Bioinformatics 21:537–539. doi:10.1093/bioinformatics/bti054. 15479716

[17] Stibitz S, Garletts TL 1992 Derivation of a physical map of the chromosome of *Bordetella pertussis* Tohama I. J Bacteriol 174:7770–7777. doi:10.1128/jb.174.23.7770-7777.1992. 1447143 PMC207492

[18] Chao MC, Abel S, Davis BM, Waldor MK 2016 The design and analysis of transposon insertion sequencing experiments. Nat Rev Microbiol 14:119–128. doi:10.1038/nrmicro.2015.7. 26775926 PMC5099075

[19] DeJesus MA, Ambadipudi C, Baker R, Sassetti C, Ioerger TR 2015 TRANSIT–a software tool for Himar1 TnSeq analysis. PLoS Comput Biol 11:e1004401. doi:10.1371/journal.pcbi.1004401. 26447887 PMC4598096

[20] Pritchard JR, Chao MC, Abel S, Davis BM, Baranowski C, Zhang YJ, Rubin EJ, Waldor MK 2014 ARTIST: high-resolution genome-wide assessment of fitness using transposon-insertion sequencing. PLoS Genet 10:e1004782. doi:10.1371/journal.pgen.1004782. 25375795 PMC4222735

[21] DeJesus MA, Ioerger TR 2013 A hidden Markov model for identifying essential and growth-defect regions in bacterial genomes from transposon insertion sequencing data. BMC Bioinformatics 14:303. doi:10.1186/1471-2105-14-303. 24103077 PMC3854130

[22] Thalen M, van den IJssel J, Jiskoot W, Zomer B, Roholl P, de Gooijer C, Beuvery C, Tramper J 1999 Rational medium design for *Bordetella pertussis*: basic metabolism. J Biotechnol 75:147–159. doi:10.1016/S0168-1656(99)00155-8. 10553654

[23] Izac M, Garnier D, Speck D, Lindley ND 2015 A functional tricarboxylic acid cycle operates during growth of *Bordetella pertussis* on amino acid mixtures as sole carbon substrates. PLoS One 10:e0145251. doi:10.1371/journal.pone.0145251. 26684737 PMC4684311

[24] Brutinel ED, Gralnick JA 2012 Anomalies of the anaerobic tricarboxylic acid cycle in *Shewanella oneidensis* revealed by Tn-seq. Mol Microbiol 86:273–283. doi:10.1111/j.1365-2958.2012.08196.x. 22925268

[25] Alonso S, Pethe K, Mielcarek N, Raze D, Locht C 2001 Role of ADP-ribosyltransferase activity of pertussis toxin in toxin-adhesin redundancy with filamentous hemagglutinin during *Bordetella pertussis* infection. Infect Immun 69:6038–6043. doi:10.1128/IAI.69.10.6038-6043.2001. 11553541 PMC98732

[26] Moon K, Bonocora RP, Kim DD, Chen Q, Wade JT, Stibitz S, Hinton DM 2017 The BvgAS regulon of *Bordetella pertussis*. mBio 8:e01526-17. doi:10.1128/mBio.01526-17. 29018122 PMC5635692

[27] van Beek LF, de Gouw D, Eleveld MJ, Bootsma HJ, de Jonge MI, Mooi FR, Zomer A, Diavatopoulos DA 2018 Adaptation of *Bordetella pertussis* to the respiratory tract. J Infect Dis 217:1987–1996. doi:10.1093/infdis/jiy125. 29528444

[28] Fernandez RC, Weiss AA 1994 Cloning and sequencing of a *Bordetella pertussis* serum resistance locus. Infect Immun 62:4727–4738. 7927748 10.1128/iai.62.11.4727-4738.1994 PMC303180

[29] Elder KD, Harvill ET 2004 Strain-dependent role of BrkA during *Bordetella pertussis* infection of the murine respiratory tract. Infect Immun 72:5919–5924. doi:10.1128/IAI.72.10.5919-5924.2004. 15385494 PMC517575

[30] Pishko EJ, Betting DJ, Hutter CS, Harvill ET 2003 *Bordetella pertussis* acquires resistance to complement-mediated killing *in vivo*. Infect Immun 71:4936–4942. doi:10.1128/iai.71.9.4936-4942.2003. 12933835 PMC187338

[31] Vanderpool CK, Armstrong SK 2001 The *Bordetella bhu* locus is required for heme iron utilization. J Bacteriol 183:4278–4287. doi:10.1128/JB.183.14.4278-4287.2001. 11418569 PMC95318

[32] Brickman TJ, Hanawa T, Anderson MT, Suhadolc RJ, Armstrong SK 2008 Differential expression of *Bordetella pertussis* iron transport system genes during infection. Mol Microbiol 70:3–14. doi:10.1111/j.1365-2958.2008.06333.x. 18554331 PMC2575024

[33] Lunak ZR, Noel KD 2015 A quinol oxidase, encoded by *cyoABCD*, is utilized to adapt to lower O2 concentrations in *Rhizobium etli* CFN42. Microbiology 161:203–212. doi:10.1099/mic.0.083386-0. 25370750 PMC4274787

[34] Oberhardt MA, Palsson BØ, Papin JA 2009 Applications of genome-scale metabolic reconstructions. Mol Syst Biol 5:320. doi:10.1038/msb.2009.77. 19888215 PMC2795471

[35] Dunphy LJ, Papin JA 2018 Biomedical applications of genome-scale metabolic network reconstructions of human pathogens. Curr Opin Biotechnol 51:70–79. doi:10.1016/j.copbio.2017.11.014. 29223465 PMC5991985

[36] Branco dos Santos F, Olivier BG, Boele J, Smessaert V, De Rop P, Krumpochova P, Klau GW, Giera M, Dehottay P, Teusink B, Goffin P 2017 Probing the genome-scale metabolic landscape of *Bordetella pertussis*, the causative agent of whooping cough. Appl Environ Microbiol 83:e01528-17. doi:10.1128/AEM.01528-17. 28842544 PMC5648915

[37] Stainer DW, Scholte MJ 1970 A simple chemically defined medium for the production of phase I *Bordetella pertussis*. J Gen Microbiol 63:211–220. doi:10.1099/00221287-63-2-211. 4324651

[38] Palmer KL, Aye LM, Whiteley M 2007 Nutritional cues control *Pseudomonas aeruginosa* multicellular behavior in cystic fibrosis sputum. J Bacteriol 189:8079–8087. doi:10.1128/JB.01138-07. 17873029 PMC2168676

[39] West NP, Jungnitz H, Fitter JT, McArthur JD, Guzmán CA, Walker MJ 2000 Role of phosphoglucomutase of *Bordetella bronchiseptica* in lipopolysaccharide biosynthesis and virulence. Infect Immun 68:4673–4680. doi:10.1128/iai.68.8.4673-4680.2000. 10899872 PMC98408

[40] Geurtsen J, Dzieciatkowska M, Steeghs L, Hamstra H-J, Boleij J, Broen K, Akkerman G, el Hassan H, Li J, Richards JC, Tommassen J, van der Ley P 2009 Identification of a novel lipopolysaccharide core biosynthesis gene cluster in *Bordetella pertussis*, and influence of core structure and lipid A glucosamine substitution on endotoxic activity. Infect Immun 77:2602–2611. doi:10.1128/IAI.00033-09. 19364841 PMC2708539

[41] Bart MJ, van Gent M, van der Heide HGJ, Boekhorst J, Hermans P, Parkhill J, Mooi FR 2010 Comparative genomics of prevaccination and modern *Bordetella pertussis* strains. BMC Genomics 11:627. doi:10.1186/1471-2164-11-627. 21070624 PMC3018138

[42] Bartell JA, Blazier AS, Yen P, Thøgersen JC, Jelsbak L, Goldberg JB, Papin JA 2017 Reconstruction of the metabolic network of *Pseudomonas aeruginosa* to interrogate virulence factor synthesis. Nat Commun 8:14631. doi:10.1038/ncomms14631. 28266498 PMC5344303

[43] de Lorenzo V 2015 *Pseudomonas aeruginosa*: the making of a pathogen. Environ Microbiol 17:1–3. doi:10.1111/1462-2920.12620. 25297499

[44] Kimura S, Hubbard TP, Davis BM, Waldor MK 2016 The nucleoid binding protein H-NS biases genome-wide transposon insertion landscapes. mBio 7:e01351-16. doi:10.1128/mBio.01351-16. 27578758 PMC4999555

[45] Sealey KL, Harris SR, Fry NK, Hurst LD, Gorringe AR, Parkhill J, Preston A 2015 Genomic analysis of isolates from the United Kingdom 2012 pertussis outbreak reveals that vaccine antigen genes are unusually fast evolving. J Infect Dis 212:294–301. doi:10.1093/infdis/jiu665. 25489002

[46] Leaché AD, Oaks JR 2017 The utility of single nucleotide polymorphism (SNP) data in phylogenetics. Annu Rev Ecol Evol Syst 48:69–84. doi:10.1146/annurev-ecolsys-110316-022645.

[47] Carlton VEH, Ireland JS, Useche F, Faham M 2006 Functional single nucleotide polymorphism-based association studies. Hum Genomics 2:391–402. doi:10.1186/1479-7364-2-6-391. 16848977 PMC3525158

