# Supplementary material for: In Vivo Gene Essentiality and Metabolism in Bordetella pertussis
Source: mSphere. 2019 May 22;4(3):e00694-18. doi: 10.1128/mSphere.00694-18 (PMC6531889; doi:10.1128/mSphere.00694-18)
Supplement: TEXT S1 [file mSphere.00694-18-s0001.docx]

**Supplemental Methods**

**Bacterial strains and growth conditions.** *B. pertussis* UT25Sm1 (1) (Genbank accession ASM168662v1) was provided by Dr. Sandra Armstrong. *B. pertussis* strains were plated on Bordet-Genou agar (BG, Gibco) containing 15% defibrinated sheep blood (Cocalico) and incubated for 48-72 hours at 37°C. Bacteria were transferred to modified synthetic Stainer-Scholte liquid media (SSM) (2, 3), when specified, and grown for 20-24 hours at 35.5°C shaking. Bacterial cultures were diluted to an OD_600_ of 0.08 and grown another 20-24 hours. Escherichia coli RHO3 (auxotroph for diaminopimelic acid (DAP) (4) carrying plasmid pSAM-Km (gift from Gerald Pier) (5) was used as the donor strain in matings and was maintained in LA or LB containing 50 μg/ml kanamycin and 400 μg/ml DAP.

**Generation of new *B. pertussis* strains.** The transposon library was created in *B. pertussis* strain UT25-*lux*. pUC18T-mini-Tn7T-lux-Gm (6) and pTNS3 (7) were transferred into *E. coli* strain RHO3 (4) by electroporation and resulting clones were maintained on LA containing antibiotics (gentamicin 20 ug/ml or carbenicillin 100 ug/ml, respectively) with 400 ug/ml DAP to maintain RHO3.  To generate *B. pertussis* strain UT25-*lux,* RHO3 pUC18T-mini-Tn7T-lux-Gm and RHO3 pTNS3 were then tri-parentally mated with *B. pertussis* strain UT25 on BG agar containing DAP 400 ug/ml for 48 hrs.  The conjugation was then swabbed on BG lacking DAP but containing gentamicin 30 ug/ml.  Gentamicin resistance luminescent colonies were then detected on a Bio-Rad XRS+ gel doc.  Clones were then isolated and PCR confirmed to contain the lux-mini-Tn7 construct on the chromosome. Additionally, a *B. pertussis* UT25 strain containing an in-frame deletion in the *bvgAS* genes was constructed by a pSS4894-based allelic exchange method using published primers and vectors for strain QC3691, which is a *B. pertussis* BP536 strain carrying *bvgAS* gene deletion (8).

**Preparation of samples for sequencing.** A 2.5 hour digestion of 3 μg of genomic DNA with 6U MmeI (New England Biolabs) was performed, and bands between 1-2kb were excised. Adapters were ligated onto the fragments overnight, and then the products were PCR purified to remove excess adapter. A PCR reaction was performed to amplify the Tn-genomic junction, add a barcode sequence, and add required Illumina sequences (P7, flow cell binding) (S8 Table). This sample was purified by size exclusion (about 125 bp). Samples were submitted to the University of Maryland Institute for Genome Sciences for quality control analysis and sequencing by Illumina HiSeq (150bp, paired-end reads). Reads were separated by barcode and then trimmed to only the 17 bp *Bordetella pertussis* genomic DNA fragment. Processing included: eliminating reads that corresponding to PhiX (loading control for Illumina), confirming that all reads contained transposon sequence and had genomic DNA starting with TA (the insertion site for mariner transposons), and that reads mapped to the *Bordetella pertussis* Tohama I genome (Genbank accession ASM19571v1). Between 35-72% of all reads per sample were included based on these parameters.

**Processing of Tn-seq reads.** Reads were trimmed to remove the transposon sequence and filtered to eliminate potential PhiX contaminants using Trimmomatic (9). Reads were then further filtered to those that were still full length following the removal of the transposon sequence, and of reads that did not either start with ‘TA’ or end with its reverse complement. These processing steps were performed with in-house shell scripts employing bioawk (<https://github.com/lh3/bioawk>).

**Conservative test of gene essentiality.** Initial analysis of *in vitro* essentiality was performed at the gene level based on the methods of Brutinal and Gralnick, which uses a series of custom bioawk commands to process the reads, map them to the reference genome, and compile hit statistics (10). A description of the pipeline can be found at (https://github.com/jbadomics/tnseq). Insertions within the last 5% of gene sequence were excluded from these analyses because they are less likely to affect gene function. The cutoffs for this analysis were stringent: an essential gene was classified as essential only if it contained zero insertions within the first 95% of gene as insertions within this region of the gene are more likely to disrupt gene function. Based on five input samples grown on BG media, the analysis identified 400 essential genes (including tRNAs and rRNAs) (S1 Table), which make up about 11.5% of the genome.

**Essentiality Analyses with TRANSIT.** In order to better utilize the statistical power enabled by the large size of our library and the number of replicates in our data set, we decided to analyze by TA insertion site rather than gene. Essentiality analysis was first performed using TRANSIT (11). The Tohama I strain of *Bordatella pertussis* was used as the reference genome. The Hidden Markov Model (HMM) mode of analysis was used to test for essential genes. TA sites within 5% of the base pair length of either the N-terminus or the C-terminus of any annotated gene were excluded from the analysis. Normalization was done using the Trimmed Total Reads (TTR) method, which normalizes by the total read-counts but trimming the top and bottom 5% of read-counts. This normalization method is recommended by the developers for most cases because it accounts for differences in saturation. Tests of conditional essentiality between sample groups was also performed using TRANSIT’s resampling test of conditional essentiality. Default settings were used for all parameters, except that the analysis was run in adaptive resampling mode for greater processing speed. The TRANSIT software tool (11) assigns each TA site a most probable essentiality state (essential, non-essential, growth-defect, and growth advantage) and classified 691 genes as essential (S2 Table), more than that identified using the stringent analysis. Whereas the stringent analysis identifies genes with any insertion as non-essential, analysis by TRANSIT identifies genes as essential based on the most represented TA site “state”, which can lead to classification of genes as essential even when a region of the gene tolerates insertion.

**Essentiality Analyses with ARTIST.** We also performed tests of essentiality and conditional essentiality using the ARTIST suite of Tn-seq analysis scripts in Matlab (12). The analysis was performed as described in the ARTIST user manual. Like TRANSIT, ARTIST uses an HMM to predict the essentiality of each gene in the reference annotation. EL-ARTIST tests for the essentiality of each gene, while CON-ARTIST tests for conditional essentiality between sample categories. The CON-ARTIST test only analyzes one sample against its paired control, so in order to determine category-wide conditional essentiality, a consensus call across samples was generated by computing the modal result across all sample pairs. ARTIST analysis is based on the sliding window method for identifying underrepresented regions followed by an HMM-refinement for making final classifications of nonessential, essential, and domain-essential genes. Using the ARTIST tool, 858 genes were classified as essential (S2 Table), and 609 of these genes were also identified using the TRANSIT pipeline (Fig 2B, S2 Table).

**Exclusion of domain-essential genes from HMM-analysis.** Through classifying the essentiality state of each TA site in a genome, HMM-based analysis of essentiality permits identification of non-gene-centric essential regions, which can be smaller (regulatory regions or individual protein domains) or larger (operons or islands of genes) than the defined coding regions. There is a context dependence of the interpretation of these results; theoretically, if part of a gene is essential, one could conclude that the entire gene should be essential. However, it is possible that the essentiality call of that region could be independent of gene function and could disrupt an unidentified regulatory region, for example a small RNA. Because of this uncertainty and need for context, we excluded the domain-essential genes identified by ARTIST from our essential gene list, but we have included them in S2 Table for future exploration of their function.

***in silico* analysis.** The two genome-scale metabolic network reconstructions (GENREs) (13, 14) were downloaded in SBML (Systems Biology Markup Language) format, loaded in MATLAB, and analyzed with the COBRA (COnstraint Based Reconstruction and Analysis) Toolbox (15, 16). The model published by Fyson *et al*. (13) contained 1153 reactions, 1092 metabolites, and 796 genes, while the model published by Dos Santos *et al.* (14) consisted of 1878 reactions, 1255 metabolites, and 770 genes. Gene essentiality for both models was tested in three different simulated media conditions: an *in vivo* mimetic sputum media (SCFM media) condition (17), SSM (2), and an alternative SSM formulation described by Dos Santos et al (14). These conditions were simulated by adjusting the model’s exchange reactions that control the import and export of particular metabolites and energy sources. The *in silico* gene essentiality predictions were compared with two experimentally generated *in vitro* lists (TRANSIT/ARTIST overlap consisting of 609 genes and conservative analysis tolerating no insertions containing 400 genes) and one *in vivo* list, all generated in this study. Single gene deletions *in silico* were performed with the Cobra Toolbox “deleteModelGenes” command (16). Genes were considered essential when the modeled organism was computationally incapable of generating biomass. The results of the computational analysis were compared to experimental datasets in order to compute the true positive, true negative, false positive, and false negative predictions, along with the accuracy, sensitivity, specificity, positive predictive value, negative predictive value, and the Matthew’s Correlation Coefficient as has been done recently for other modeled organisms (18, 19) (S7 Table). For particular genes of interest, the mechanism of their essentiality was explored by computationally determining the metabolites that were unable to be synthesized as a result of the absence of function associated with the gene. This analysis provided insight into the biological significance of certain genes necessary for growth.

**References**

1. Parker CD, Doyle S, Field LH, Hewlett E. 1980. Variability in derivative strains of Bordetella pertussis. Dev Biol Stand 45:119–127.

2. Stainer DW, Scholte MJ. 1970. A simple chemically defined medium for the production of phase I Bordetella pertussis. J Gen Microbiol 63:211–220.

3. Hewlett E, Wolff J. 1976. Soluble adenylate cyclase from the culture medium of Bordetella pertussis: purification and characterization. J Bacteriol 127:890–898.

4. López CM, Rholl DA, Trunck LA, Schweizer HP. 2009. Versatile dual-technology system for markerless allele replacement in Burkholderia pseudomallei. Appl Environ Microbiol 75:6496–6503.

5. Skurnik D, Roux D, Aschard H, Cattoir V, Yoder-Himes D, Lory S, Pier GB. 2013. A comprehensive analysis of in vitro and in vivo genetic fitness of Pseudomonas aeruginosa using high-throughput sequencing of transposon libraries. PLoS Pathog 9:e1003582.

6. Damron FH, McKenney ES, Barbier M, Liechti GW, Schweizer HP, Goldberg JB. 2013. Construction of mobilizable mini-Tn7 vectors for bioluminescent detection of gram-negative bacteria and single-copy promoter lux reporter analysis. Appl Environ Microbiol 79:4149–4153.

7. Choi K-H, Mima T, Casart Y, Rholl D, Kumar A, Beacham IR, Schweizer HP. 2008. Genetic Tools for Select-Agent-Compliant Manipulation of Burkholderia pseudomallei. Appl Environ Microbiol 74:1064–1075.

8. Chen Q, Ng V, Warfel JM, Merkel TJ, Stibitz S. 2017. Activation of Bvg-Repressed Genes in Bordetella pertussis by RisA Requires Cross Talk from Noncooperonic Histidine Kinase RisK. J Bacteriol 199.

9. Bolger AM, Lohse M, Usadel B. 2014. Trimmomatic: a flexible trimmer for Illumina sequence data. Bioinforma Oxf Engl 30:2114–2120.

10. Brutinel ED, Gralnick JA. 2012. Anomalies of the anaerobic tricarboxylic acid cycle in Shewanella oneidensis revealed by Tn-seq. Mol Microbiol 86:273–283.

11. DeJesus MA, Ambadipudi C, Baker R, Sassetti C, Ioerger TR. 2015. TRANSIT--A Software Tool for Himar1 TnSeq Analysis. PLoS Comput Biol 11:e1004401.

12. Pritchard JR, Chao MC, Abel S, Davis BM, Baranowski C, Zhang YJ, Rubin EJ, Waldor MK. 2014. ARTIST: high-resolution genome-wide assessment of fitness using transposon-insertion sequencing. PLoS Genet 10:e1004782.

13. Fyson N, King J, Belcher T, Preston A, Colijn C. 2017. A curated genome-scale metabolic model of Bordetella pertussis metabolism. PLoS Comput Biol 13:e1005639.

14. Branco Dos Santos F, Olivier BG, Boele J, Smessaert V, De Rop P, Krumpochova P, Klau GW, Giera M, Dehottay P, Teusink B, Goffin P. 2017. Probing the genome-scale metabolic landscape of Bordetella pertussis, the causative agent of whooping cough. Appl Environ Microbiol.

15. Schellenberger J, Que R, Fleming RMT, Thiele I, Orth JD, Feist AM, Zielinski DC, Bordbar A, Lewis NE, Rahmanian S, Kang J, Hyduke DR, Palsson BØ. 2011. Quantitative prediction of cellular metabolism with constraint-based models: the COBRA Toolbox v2.0. Nat Protoc 6:1290–1307.

16. Becker SA, Feist AM, Mo ML, Hannum G, Palsson BØ, Herrgard MJ. 2007. Quantitative prediction of cellular metabolism with constraint-based models: the COBRA Toolbox. Nat Protoc 2:727–738.

17. Palmer KL, Aye LM, Whiteley M. 2007. Nutritional Cues Control Pseudomonas aeruginosa Multicellular Behavior in Cystic Fibrosis Sputum. J Bacteriol 189:8079–8087.

18. Heavner BD, Price ND. 2015. Comparative Analysis of Yeast Metabolic Network Models Highlights Progress, Opportunities for Metabolic Reconstruction. PLOS Comput Biol 11:e1004530.

19. Kavvas ES, Seif Y, Yurkovich JT, Norsigian C, Poudel S, Greenwald WW, Ghatak S, Palsson BO, Monk JM. 2018. Updated and standardized genome-scale reconstruction of Mycobacterium tuberculosis H37Rv, iEK1011, simulates flux states indicative of physiological conditions. BMC Syst Biol 12:25.
