# Supplementary figures and images for: In Vivo Gene Essentiality and Metabolism in Bordetella pertussis
Source: mSphere. 2019 May 22;4(3):e00694-18. doi: 10.1128/mSphere.00694-18 (PMC6531889; doi:10.1128/mSphere.00694-18)

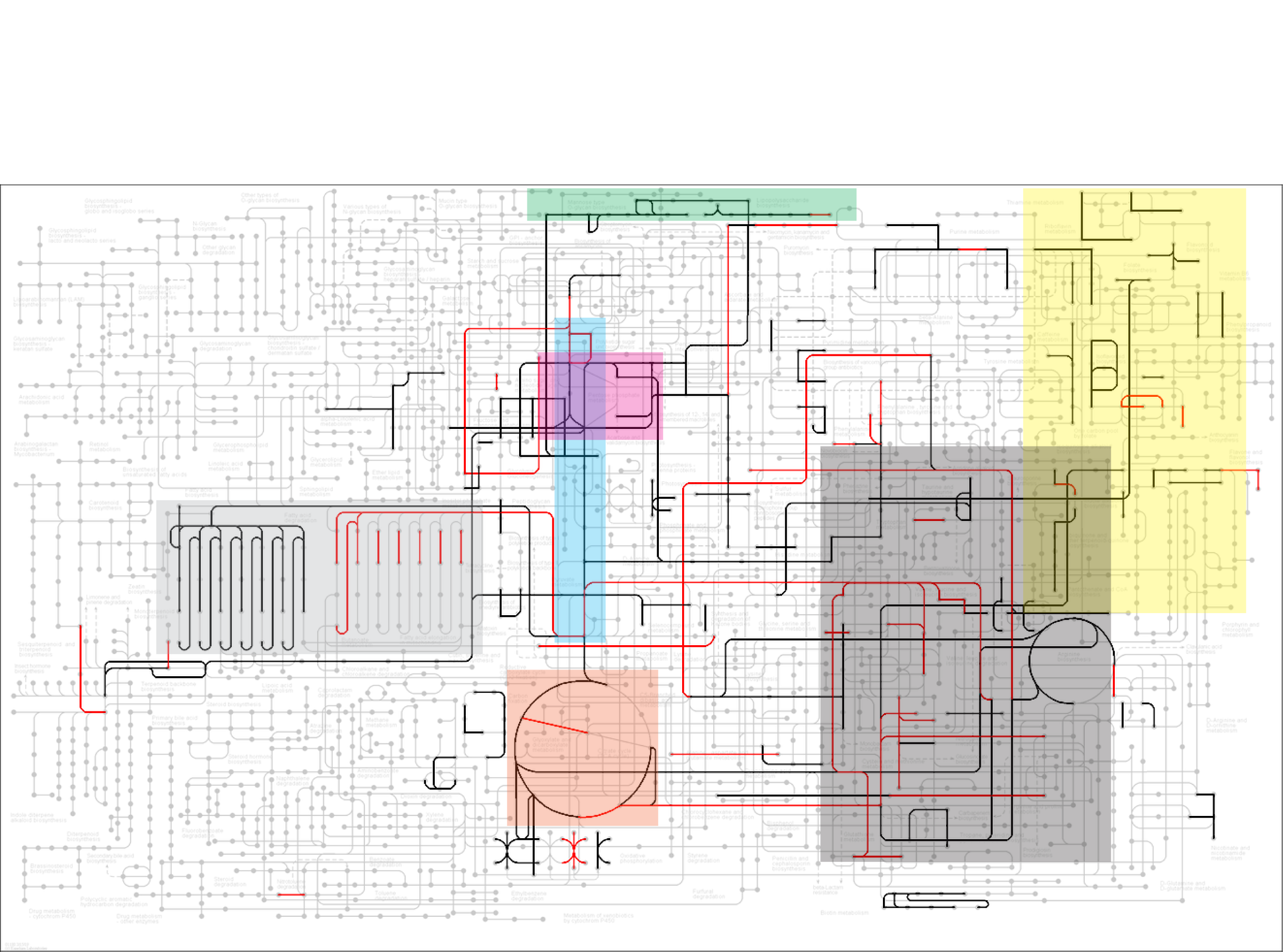

Supplement: FIG S1 [file mSphere.00694-18-sf001.tif]

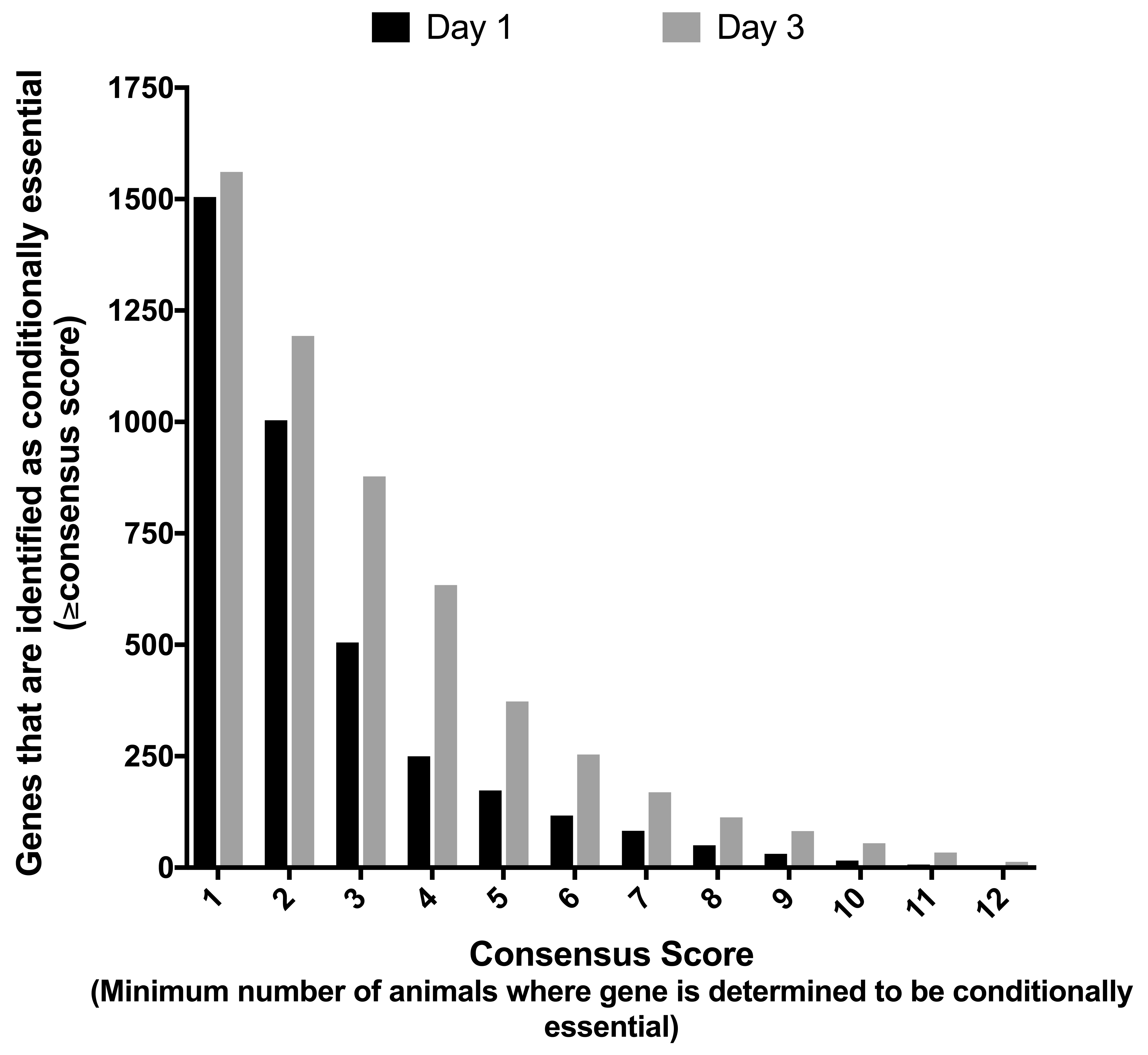

Supplement: FIG S2 [file mSphere.00694-18-sf002.tif]
